# Perceived memory reliability and music performance anxiety in Chinese musicians: a mediation and latent profile approach

**DOI:** 10.3389/fpsyg.2025.1724226

**Published:** 2026-01-02

**Authors:** Jing Li, Yingli Luo, Zaihao Wu, Lijun Huang, Jian Sun

**Affiliations:** 1 School of Music and Dance, Xihua University, Chengdu, China; 2 Chengdu Zhonghe Vocational Middle School, Chengdu, China

**Keywords:** Chinese musicians, latent profile analysis, mediation, music performance anxiety, perceived memory reliability, performance worry

## Abstract

**Introduction:**

The present study aimed to clarify heterogeneity in music performance anxiety (MPA) by identifying latent profiles, examining sociodemographic and psychological predictors of profile membership, and testing mediation pathways.

**Methods:**

A total of 819 Chinese musicians participated in an online survey that assessed MPA, performance worry (PW), and perceived memory reliability (PMR), along with demographic variables.

**Results:**

Latent profile analysis (LPA) revealed a three-profile solution that distinguished low, moderate, and high MPA groups. Multinomial logistic regression indicated that older age, higher education levels, lower household income, and unstable employment were significantly associated with membership in the moderate and high MPA profiles. In addition, PW emerged as a significant psychological predictor of elevated MPA, whereas PMR showed a protective effect and was negatively associated with MPA. Mediation models further demonstrated that PW played an important role in transmitting the effect of PMR on MPA, suggesting that cognitive factors related to memory reliability shape worry processes, which, in turn, intensify performance anxiety.

**Conclusions:**

These findings advance understanding of MPA by demonstrating that Chinese musicians can be meaningfully categorized into distinct risk groups, each shaped by sociodemographic vulnerabilities and cognitive-emotional pathways. From a practical perspective, the results highlight the importance of targeted prevention and intervention strategies that address both memory-related cognitions and performance worry in order to reduce MPA in vulnerable populations.

## 1 Introduction

Music Performance Anxiety (MPA) is a widespread phenomenon among both professional and student musicians (Kenny, 2023; Papageorgi, 2020). It refers to a constellation of adverse physiological, cognitive, and behavioral reactions that arise in anticipation of or during performance situations. Typical manifestations include palpitations, excessive sweating, difficulties in sustaining attention, muscle tension, and, in more severe cases, active avoidance of performance opportunities (de Lima et al., 2024). Epidemiological data suggest that a substantial proportion of musicians experience MPA at levels that interfere with performance quality, career development, and psychological wellbeing (Zhang and Jenatabadi, 2024). High MPA has been linked to impaired concentration, increased memory slips, reduced expressive quality, and even premature termination of musical careers (Gómez-López and Sánchez-Cabrero, 2023; Kinney et al., 2025). Furthermore, comorbid conditions such as depression, generalized anxiety, and burnout frequently co-occur, indicating that MPA is not only a situational challenge but also a broader health concern (Wang and Yang, 2024). Given the dual impact on performance outcomes and mental health, understanding the cognitive and emotional mechanisms underlying MPA has become an urgent research priority.

An important but relatively understudied factor in MPA is perceived memory reliability (PMR), which can be understood as the subjective confidence musicians hold regarding the accuracy, stability, and accessibility of their memory during performance (Kenny, 2023). Musical performance frequently requires extended passages to be delivered entirely from memory, and even minor lapses are highly salient, often disproportionately emphasized by both performers and audiences. Consequently, self-evaluations of memory reliability form a critical dimension of cognitive appraisal under pressure (Ou and Qin, 2025; Wang and Yang, 2024). When PMR is low, performers may perceive reduced control over their execution, a pattern consistent with Bandura’s framework of self-efficacy (Bandura, 2001), wherein diminished confidence in task-relevant capacities heightens susceptibility to anxiety (Lu et al., 2024b). In contrast, high PMR can foster a sense of mastery and resilience, buffering against worry and performance disruption (Hu, 2024; MacAfee and Comeau, 2020). Although previous research has examined predictors of MPA such as personality traits, technical competence, and practice routines (de Lima et al., 2024), cognitive self-appraisals like PMR have received comparatively less empirical attention. Thus, clarifying the role of PMR on MPA therefore addresses a significant gap.

Performance worry (PW) may serve as a plausible mediator linking PMR to MPA. PW refers to anticipatory concerns that take the form of repetitive and intrusive thoughts about potential failure, negative evaluation, or loss of control during performance (Borkovec et al., 1998; Kenny, 2011). When PMR is low, performers are more likely to engage in excessive self-monitoring and to worry about possible memory lapses, a process that consumes attentional resources, heightens physiological arousal, and ultimately undermines concentration and execution (Angelidis et al., 2019; Held et al., 2020). This mechanism is consistent with Attentional Control Theory, which proposes that worry redirects attention away from task-relevant goals and toward threat-related cues, thereby reducing performance efficiency (Eysenck et al., 2007). This pathway also aligns with broader principles of self-efficacy theory, which links lower confidence in one’s capabilities to heightened vulnerability to worry-driven anxiety responses (Bandura, 2001). Although empirical research has not yet directly tested PW as a mediator between PMR and MPA, several studies provide indirect support for this pathway. In academic settings, worry has been shown to mediate the association between negative affect and impaired performance, illustrating how self-doubt can translate into distress through worry processes (Owens et al., 2012). Within music contexts, low self-efficacy has been linked to stronger performance-related concerns that correlate with higher levels of MPA (Wang and Yang, 2024). Structural modeling studies have further demonstrated that self-efficacy mediates the effect of perfectionistic tendencies on MPA, highlighting the important role of cognitive self-evaluations in activating performance-related worry (Muthuswamy and Ragavendran, 2024). In sum, these findings suggest that while the mediating role of PW requires more direct empirical examination, there is substantial indirect evidence supporting its function as a cognitive mechanism through which low PMR heightens vulnerability to MPA.

Although variable-centered models clarify directional pathways, the effects of PMR and PW on MPA are unlikely to be uniform across all musicians. Both the direct effect of PMR and the indirect effect through PW may differ across groups characterized by distinct levels of MPA. This possibility highlights the value of Latent Profile Analysis (LPA), a person-centered approach that can identify subgroups of individuals based on their MPA profiles. In music education, He and Chen (2024) employed LPA with pre-service music teachers and identified four profiles defined by perfectionism and control-value beliefs, which were associated with markedly different levels of MPA. Their results illustrate that psychological risk factors exert unequal effects across subpopulations, highlighting the need to account for heterogeneity. Yet research applying LPA directly to MPA remains scarce. The present study addresses this gap by applying LPA to identify subgroups of musicians differing not only in their MPA levels but also in the strength of the PMR-PW-MPA pathway. By integrating both perspectives, this study examines whether these cognitive mechanisms operate differently across naturally occurring MPA subgroups.

Building on this rationale, the present study applies LPA directly to MPA while simultaneously testing mediation pathways involving PMR and PW. This integrated design captures both generalizable mechanisms and subgroup heterogeneity. From a variable-centered perspective, the mediation model evaluates whether PW constitutes the principal pathway through which PMR influences performance anxiety. From a person-centered perspective, LPA identifies distinct MPA profiles and examines whether the strength of these mediation pathways differs across subgroups. These approaches situate PMR and PW within a coherent cognitive–emotional framework of MPA without reiterating full theoretical descriptions. Beyond theoretical contributions, this dual framework provides practical implications for educators and performers, offering evidence to guide interventions that aim to enhance memory confidence, reduce worry, and ultimately alleviate MPA.

In light of these considerations, the present study pursues two primary aims. First, it applies LPA to classify musicians into subgroups with distinct levels of MPA, thereby capturing heterogeneity in how MPA manifests across individuals. Second, it examines the mediating role of PW in the relationship between PMR and MPA, and tests whether this mechanism operates consistently across the identified profiles. We hypothesize that low PMR will be associated with higher PW, which in turn predicts greater MPA. By combining person-centered and variable-centered approaches, this study clarifies both the subgroup-specific patterns and the general mechanisms through which memory-related self-appraisals influence performance anxiety.

## 2 Methods

### 2.1 Participants and data collection

Participants were recruited from five community-based choirs in the east of China. Data collection took place between July and August 2025 using an online survey platform that ensures secure and anonymous data collection. Eligibility criteria required that participants were active choir members aged 18 years or above and had participated in at least one public performance during the preceding 12 months, thereby ensuring sufficient engagement in music performance activities. Individuals who reported severe mental illness or cognitive impairment were excluded to minimize confounding influences on performance-related psychological variables. After applying these criteria and removing invalid responses based on attention checks, a total of 819 participants were included in the final sample.

Prior to participation, all respondents were informed of the study’s purpose, procedures, and their rights as participants. Electronic informed consent was obtained, and confidentiality was strictly maintained throughout the process. Participation was entirely voluntary, and no financial or material incentives were offered. To ensure data quality, attention-check items and logic consistency checks were embedded within the survey, and responses failing these criteria were excluded from the final dataset. The study protocol was reviewed and approved by the Ethics Committee of Xihua University (Approval No. 250626-01).

### 2.2 Measures

#### 2.2.1 Background factors

Background factors included participants’ age, sex, monthly household income, and employment status.

#### 2.2.2 MPA

MPA was assessed using the Chinese version of the Stage Music Performance Anxiety Inventory (Su et al., 2017). This instrument consists of 14 items that measure anxious responses in actual performance settings. A sample item includes “I feel nervous” and “I worry that today’s performance may go wrong.” All items are rated on a seven-point Likert scale ranging from 1 (Never) to 7 (Always), with higher scores indicating greater levels of performance anxiety. In the present study, the Cronbach’s alpha was 0.95, indicating excellent internal consistency.

#### 2.2.3 PW

PW was assessed using the Worry scale (de Paula Ortiz et al., 2023). This subscale captures musicians’ negative cognitions, worries, and catastrophic thoughts during performance situations (e.g., “Thoughts about possible evaluations interfere with my performance”; “I often prepare for concerts with a sense of dread and impending disaster”). Each item was rated on a seven-point Likert scale ranging from 1 (Never) to 7 (Always), with higher scores indicating greater levels of performance-related worry. In the present study, it demonstrated good internal consistency, with a Cronbach’s alpha of 0.93.

#### 2.2.4 PMR

A two-item Memory scale was used (Kenny, 2023). This brief scale has been used in previous studies with musicians and has shown satisfactory psychometric properties. A two-item format was adopted because PMR represents a highly specific appraisal—confidence in recalling memorized material during performance—where a concise measure can effectively capture the construct while minimizing participant burden. The items were: “I often feel that my memory is unreliable when playing without a score” and “I lack confidence in performing from memory.” Items were rated on a seven-point Likert scale ranging from 1 (Strongly disagree) to 7 (Strongly agree). Because both items are negatively worded, they were reverse-coded prior to analysis so that higher scores reflect greater perceived memory reliability. In the present study, this scale demonstrated acceptable internal consistency, with a Cronbach’s alpha of 0.90.

### 2.3 Data analysis

Statistical analyses were conducted using SPSS version 27.0 and Mplus version 8.3. First, descriptive statistics were calculated to summarize the background factors of the sample in SPSS 27.0. Second, latent profile analyses (LPA) were conducted in MPlus 8.3 to identify subgroups of participants based on their responses to the Stage Music Performance Anxiety Inventory. Competing models with increasing numbers of profiles (two-profile to five-profile solutions) were estimated and compared. Model evaluation relied on multiple statistical fit indices, including the Akaike Information Criterion (AIC), Bayesian Information Criterion (BIC), sample-size adjusted BIC (aBIC), entropy, Lo–Mendell–Rubin adjusted likelihood ratio test (LMR-LRT), and Bootstrap Likelihood Ratio Test (BLRT). Lower values of AIC, BIC, and aBIC, higher entropy values (closer to 1.0), and significant LMR-LRT and BLRT results were used to determine the optimal number of profiles, together with theoretical interpretability and parsimony (Spurk et al., 2020). In addition, each latent profile was required to include at least 5% of the total sample to ensure sufficient profile size and stability of the solution (Spurk et al., 2020).

Third, the identified MPA profiles were used as categorical dependent variables in a series of multinomial logistic regression analyses performed in SPSS 27.0. Background variables, PW, and PMR were entered as predictors to examine their associations with MPA profile membership. Finally, mediation analyses were conducted separately within each MPA profile to test whether PW mediated the association between PMR and MPA. Bootstrapping with 5,000 resamples was applied to estimate indirect effects and their 95% confidence intervals. Mediation analyses were conducted in SPSS using the PROCESS macro (Model 4; Hayes, 2018). Standardized coefficients (*β*) were reported. All statistical tests were two-tailed, with a significance level set at *p* < 0.05.

## 3 Results

### 3.1 Descriptive statistics

As presented in Table 1, the majority of participants were female (73.1%). More than half of the respondents (53.7%) reported holding a four-year university degree. With respect to monthly household income, 22.0% indicated a monthly personal income below 4,000 Yuan, whereas 11.7% reported a household income exceeding 10,000 Yuan. Regarding occupational status, 26.7% of participants were employed full time.

**Table 1:** Descriptive analysis.

Variable	*n*/mean	%/SD
Age	47.25	17.88
Sex		
Female	220	26.9
Male	599	73.1
Educational level		
High school or below	145	17.7
Three-year college	142	17.3
University	440	53.7
Master or above	92	11.2
Monthly household income		
<4,000 Yuan	180	22.0
4,000–5,999 Yuan	131	16.0
6,000–7,999 Yuan	97	11.8
8,000–9,999 Yuan	58	7.1
>10,000 Yuan	96	11.7
Choosing not to report	257	31.4
Employment status		
Students	180	22.0
Full-time	219	26.7
Unemployed	36	4.4
Choosing not to report	384	46.9

### 3.2 The latent profile of MPA

Latent profile analysis (LPA) was conducted using the 14 items of MPA as indicators. Model fit indices were evaluated for solutions with two to five profiles (Table 2). The three-profile solution showed the highest entropy value (0.955). The AIC, BIC, and adjusted BIC values decreased as the number of profiles increased. Both the Lo–Mendell–Rubin test (*p* = 0.001) and the bootstrap likelihood ratio test (*p* < 0.001) indicated that the three-profile solution provided a significantly better fit than the two-profile model. In addition, the three-profile model met the criterion that each profile contained more than 5% of the total sample. These results suggested that the three-profile solution was the most appropriate.

**Table 2:** Goodness-of-Fit indices for MPA profile.

Profile	AIC	BIC	aBIC	Entropy	LMR	BLRT	Proportion of individuals in each profile (%)
1-profile	41831.069	41962.895	41873.978	—	—	—	—
2-profile	36938.799	37141.247	37004.695	0.933	<0.001	<0.001	59.5/40.5
**3-profile**	**35238.832**	**35511.901**	**35327.715**	**0.955**	**0.001**	**<0.001**	**51.0/35.5/13.4**
4-profile	34371.875	34715.565	34483.745	0.926	0.004	<0.001	37.7/34.0/23.9/4.4
5-profile	34029.854	34444.165	34164.711	0.920	0.454	<0.001	36.4/7.6/30.5/21.1/4.4

MPA, music performance anxiety. The bold indicates the best solution.

**Figure 1:**
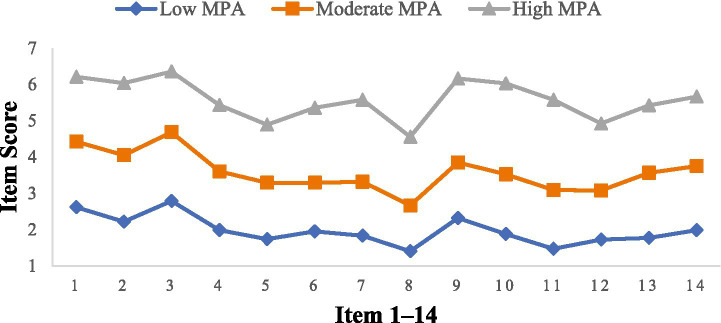
Probabilities of MPA for three latent profiles.

Figure 1 illustrates the three latent profiles. The first profile showed the lowest mean scores across all items and was labeled as low MPA (51.0%). The second profile had moderate scores on all items and was labeled as moderate MPA (35.5%). The third profile displayed the highest scores on all items and was labeled as high MPA (13.4%).

### 3.3 Predictors of MPA

Multinomial logistic regression was conducted with the low MPA profile as the reference group. The results are presented in Table 3. Age was a significant predictor, with younger participants more likely to belong to the moderate [OR = 0.96, 95% CI (0.95, 0.97)] and high MPA profiles [OR = 0.93, 95% CI (0.92, 0.94)]. For educational level, participants with a university degree were more likely to belong to the high MPA profile compared to those with high school or below [OR = 3.25, 95% CI (1.60, 6.61)]. Household income showed a consistent protective effect. Compared with those earning less than 4,000 Yuan per month, participants in higher income groups had lower odds of being in the moderate and high MPA profiles. For example, those earning more than 10,000 Yuan had a substantially lower likelihood of belonging to the high MPA profile [OR = 0.13, 95% CI (0.05, 0.35)]. Employment status was also significant. Relative to students, full-time workers [OR = 0.19, 95% CI (0.11, 0.33)] and unemployed participants [OR = 0.07, 95% CI (0.02, 0.31)] were less likely to be classified in the high MPA profile. However, Sex was not a significant predictor of profile membership.

**Table 3:** Multinomial logistic regression results predicting latent profile membership.

Predictor	Moderate MPA		High MPA	
	OR	95% CI	OR	95% CI
Age	0.96***	(0.95, 0.97)	0.93***	(0.92, 0.94)
Sex				
Male	Reference		Reference	
Female	0.82	(0.58, 1.16)	1.28	(0.82, 2.02)
Educational level				
High school or below	Reference		Reference	
Three-year college	0.57*	(0.34, 0.96)	1.04	(0.43, 2.52)
University	1.44	(0.96, 2.15)	3.25**	(1.60, 6.61)
Master or above	0.80	(0.45, 1.43)	2.05	(0.84, 5.02)
Monthly household income				
<4,000 Yuan	Reference		Reference	
4,000–5,999 Yuan	0.48**	(0.29, 0.79)	0.28**	(0.14, 0.58)
6,000–7,999 Yuan	0.44**	(0.25, 0.76)	0.26**	(0.11, 0.57)
8,000–9,999 Yuan	0.38**	(0.19, 0.75)	0.38*	(0.16, 0.91)
>10,000 Yuan	0.38**	(0.22, 0.66)	0.13***	(0.05, 0.35)
Choosing not to report	0.59*	(0.38, 0.90)	0.45**	(0.26, 0.78)
Employment status				
Students	Reference		Reference	
Full-time	0.32***	(0.20, 0.51)	0.19***	(0.11, 0.33)
Unemployed	0.29**	(0.13, 0.64)	0.07***	(0.02, 0.31)
Choosing not to report	0.18***	(0.12, 0.28)	0.06***	(0.03, 0.11)
PMR	0.64***	(0.58, 0.70)	0.85***	(0.78, 0.92)
PW	1.28***	(1.23, 1.32)	1.60***	(1.50, 1.71)

*p < 0.05, **p < 0.01, ***p < 0.001, Reference, reference group in each categorical variable; PMR, perceived memory reliability, PW, performance worry.

Regarding main variables, psychological factors strongly predicted profile membership. Higher levels of PMR decreased the odds of being in both the moderate [OR = 0.64, 95% CI (0.58, 0.70)] and high MPA profiles [OR = 0.85, 95% CI (0.78, 0.92)]. In contrast, greater PW was associated with higher odds of belonging to the moderate [OR = 1.28, 95% CI (1.23, 1.32)] and high MPA profiles [OR = 1.60, 95% CI (1.50, 1.71)].

### 3.4 Mediation effects based on MPA profiles

**Figure 2:**
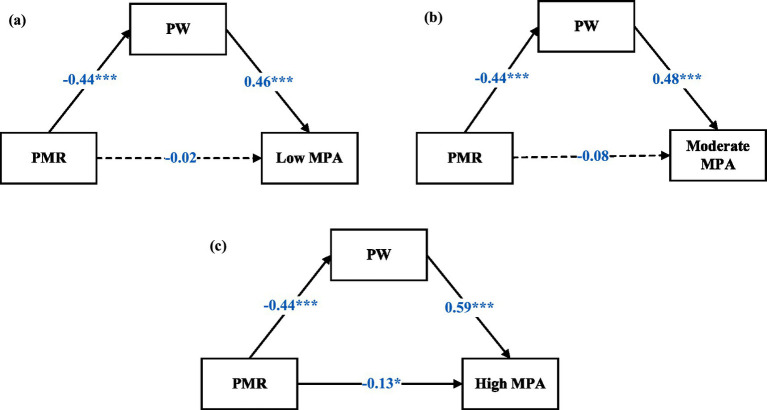
Mediation models based on MPA profiles. **(a)** Low MPA profile, **(b)** moderate MPA profile, and **(c)** high MPA profile. PMR, perceived memory reliability; PW, performance worry; MPA, music performance anxiety. *p < 0.05, ***p < 0.001. Standardized coefficients (*β*) were reported.

Figure 2 demonstrates the associations among PMR, PW, and each MPA profile. Across the three profiles, PMR had a consistent negative effect on PW (*β* = −0.44, *p* < 0.001 in all profiles). PW, in turn, predicted higher MPA, with coefficients increasing from the low MPA group (*β* = 0.46, *p* < 0.001) to the moderate MPA group (*β* = 0.48, *p* < 0.001) and the high MPA group (*β* = 0.59, *p* < 0.001). The direct path from PMR to MPA was not significant in the low MPA group (*β* = −0.02) and the moderate MPA group (*β* = −0.08), but was small and significant in the high MPA group (*β* = −0.13, *p* = 0.035). These patterns indicated that PW fully mediated the association between PMR and MPA in the low and moderate profiles, while mediation was partial in the high MPA profile, where both indirect and direct effects were present.

The bootstrapped mediation analyses further confirmed these patterns. In the low MPA profile, the indirect effect of PMR on MPA via PW was significant [*β* = −0.20, 95% CI (−0.26, −0.15)], with the direct effect remaining nonsignificant, indicating full mediation. In the moderate MPA profile, the indirect effect was also significant [*β* = −0.21, 95% CI (−0.27, −0.16)], again supporting full mediation. In the high MPA profile, the indirect effect was stronger [*β* = −0.26, 95% CI (−0.34, −0.20)], and given the presence of a small yet significant direct effect, this pattern suggested partial mediation.

## 4 Discussion

The present study aimed to clarify heterogeneity in MPA by identifying latent profiles, examining predictors of profile membership, and testing mediation pathways. Using LPA, we found a three-profile solution that distinguished low, moderate, and high MPA groups. Multinomial logistic regression showed that age, education, monthly household income, employment status, PW, and PMR were significant predictors of membership in the moderate and high MPA profiles. Mediation models further indicated that PW mediated the pathway from PMR to MPA among Chinese musicians. These results provide both conceptual and practical insights into the structure and correlates of MPA.

The present study identified three distinct profiles of MPA: low, moderate, and high. This result highlights the heterogeneous nature of MPA and suggests that performers do not experience anxiety in a uniform way. Research using latent profile analysis (LPA) in this field is still limited, with only a few studies applying person-centered methods to understand subgroup patterns. For instance, a recent study identified four profiles among Chinese pre-service music teachers, i.e., maladaptive, average, low-medium, and adaptive, and found clear differences in MPA across these groups (He and Chen, 2024). Although our study produced three severity-based profiles while theirs revealed four profiles shaped by perfectionism and control-value beliefs, both point to the value of LPA in uncovering meaningful heterogeneity. These findings suggest that MPA should not be treated as a single continuum but rather as a set of qualitatively distinct experiences. Given the small number of studies using LPA in this area, our results contribute to the growing evidence that person-centered approaches provide richer insights than traditional variable-centered methods. Future research could extend this work by integrating both symptom indicators and cognitive-motivational factors, thereby capturing not only the severity of anxiety but also the underlying mechanisms that distinguish subgroups.

The multinomial regression provided important insights into the sociodemographic factors that influence the likelihood of belonging to higher MPA profiles. Our study indicated that younger participants were more likely to fall into the moderate or high MPA groups. This finding is consistent with developmental models suggesting that younger performers have less experience in managing evaluation and may be more sensitive to external judgment (Papageorgi, 2020). Sex did not significantly predict profile membership in this sample, which contrasts with previous studies where women often reported higher MPA (Barros et al., 2024; Butković et al., 2021; Lu et al., 2024a). This indicates that equal attention to both groups is needed in research and practice. Education also showed a differentiated pattern. In the moderate MPA group, participants with a three-year college degree had lower odds than those with high school education or below, whereas university and higher degrees were not significant. In contrast, in the high MPA group, those with a four-year university degree were more likely to belong to this profile compared with those with high school or below, while three-year college, master’s, and above were not significantly different from the reference group. These results suggest that education may play a double role: certain college programs may provide performers with practical skills and confidence that help buffer against moderate anxiety, while the higher academic and evaluative demands of four-year university programs may place students under greater competitive pressure and self-expectation (Blair and Van Der Sluis, 2022; Miksza et al., 2021), thereby elevating the risk for MPA.

Self-reported household income level and employment status further shaped the likelihood of belonging to higher MPA profiles. Higher monthly income was consistently associated with lower odds of being in the moderate or high groups, which may reflect greater access to resources such as private coaching, practice facilities, and more stable living conditions that reduce stress (Rakesh et al., 2025). This pattern also aligns with previous research showing that individuals with higher socioeconomic status generally report lower levels of anxiety (Shahbazi et al., 2022). Employment status also showed a clear effect. Students were more likely to be in the higher MPA profiles than full-time workers or unemployed individuals, likely because students are more frequently exposed to auditions, examinations, and other evaluative performances that intensify anticipatory worry (de Bie et al., 2024). These findings emphasize that sociodemographic factors interact with educational and contextual conditions to shape the risk of elevated MPA, underscoring the importance of considering demographic variable when interpreting MPA profiles.

Psychological variables (PMR and PW) played important roles in differentiating profile membership. PW showed a strong positive association with belonging to the moderate and high MPA profiles, whereas PMR functioned as a protective factor, demonstrating a negative association with higher anxiety groups. Performers who doubted their ability to reliably recall prepared material, or who frequently worried about forthcoming performances, were more likely to experience heightened MPA, which aligns with self-efficacy theory (Bandura, 2001). PMR can thus be understood as a domain-specific indicator of self-efficacy, reflecting the extent to which individuals trust their memory in demanding contexts (Chaffin et al., 2016; Zelenak, 2024). Consistent with Attentional Control Theory (Eysenck et al., 2007), worry imposes a cognitive load that reduces attentional efficiency and increases sensitivity to potential threats. Within musical performance, concerns about mistakes or negative evaluation divert attention away from task goals, sustain physiological arousal, and intensify anxiety. Low PMR may amplify this process by fostering intrusive concerns that perpetuate cycles of worry, thereby reinforcing high levels of performance anxiety (Eysenck et al., 2007). These findings suggest that PMR and PW function as interconnected mechanisms linking cognitive self-appraisals to elevated MPA.

The mediation analyses clarified the mechanisms linking beliefs about memory and MPA. Across all profiles, PMR predicted higher PW, which in turn predicted higher anxiety. In the low and moderate MPA groups, this relationship was fully mediated by worry, indicating that concerns about memory increase anxiety mainly through the worry pathway. In the high MPA group, a small but significant direct link from memory beliefs to anxiety remained, suggesting partial mediation. This implies that in highly anxious performers, memory concerns may influence anxiety both by increasing worry and by directly raising physiological arousal or self-focused attention. These findings highlight the mediating role of PW. Worry acts not only as an emotional response but also as a mechanism that links cognitive appraisals to anxiety outcomes. Interventions that reduce worry, such as cognitive restructuring, mindfulness, and metacognitive therapy, may therefore be especially effective (Khoury et al., 2015). At the same time, beliefs about memory deserve attention, particularly in highly anxious performers (Williamon, 2004). Addressing unrealistic expectations about flawless recall and teaching strategies for recovery from memory slips may reduce the direct impact of memory concerns on anxiety.

The present study has several practical implications for supporting Chinese musicians. Low MPA individuals, who do not experience MPA or only mild levels of MPA, may not require intervention. For those in the moderate MPA group, practical workshops that focus on relaxation skills, stage preparation, and worry management could be beneficial (Nicholl and Abbott, 2025; Osborne et al., 2014). Such programs can be integrated into music training curricula so that musicians gradually develop strategies to regulate MPA before it becomes severe. High MPA individuals, however, may need more structured psychological support (Lu et al., 2025). Cognitive-behavioral approaches that address negative thinking, as well as guided exposure to performance settings, could help them build tolerance for stress (Osborne et al., 2007). It is also important to address beliefs about memory. Many musicians worry excessively about memory lapses; therefore, teaching strategies for error recovery and emphasizing expressive communication over perfect recall may reduce MPA (Gómez-López and Sánchez-Cabrero, 2023). Music teachers and mentors can further contribute by giving constructive feedback that encourages confidence and growth, rather than reinforcing fear of mistakes.

Several limitations should be acknowledged. First, the cross-sectional design does not establish causality. Future longitudinal studies are needed to test whether changes in memory beliefs and PW lead to changes in MPA over time. Second, all data were self-reported, raising concerns about reporting bias and shared method variance. Future research should include behavioral or physiological measures of MPA, such as heart rate, cortisol levels, or observer ratings during performance, to provide a more objective assessment. Third, although entropy was high and profile sizes acceptable, latent profile solutions can be sample-specific. Replication across diverse samples and the use of latent transition analysis would help examine whether individuals shift between profiles over time and identify factors that predict improvement or worsening. Finally, although PMR was reverse-coded to ensure consistent interpretation, future studies may benefit from more elaborated scales that distinguish between different facets of memory confidence.

## 5 Conclusion

In conclusion, MPA in this sample formed three distinct profiles representing low, moderate, and high levels of performance anxiety. Demographic factors such as age, education level, self-reported household income, and employment status helped explain who was more likely to belong to each profile. Psychological factors showed even stronger associations, as lower PMR and higher PW were both linked to increased likelihood of being in the moderate and high profiles. The mediation models further indicated that PW serves as a key pathway through which memory beliefs influence performance anxiety, with this effect being particularly pronounced among individuals in the high MPA group. These findings highlight important intervention targets that are both testable and practical, including strategies to manage worry and to reframe maladaptive beliefs about memory. By integrating person-centered modeling with evidence on cognitive mechanisms, the study provides a foundation for developing more precise and effective approaches to support musicians who experience elevated MPA.

## Data Availability

The raw data supporting the conclusions of this article will be made available by the authors, without undue reservation.
